# Brain and Nasal Cavity Anatomy of the Cynomolgus Monkey: Species Differences from the Viewpoint of Direct Delivery from the Nose to the Brain

**DOI:** 10.3390/pharmaceutics12121227

**Published:** 2020-12-18

**Authors:** Toshiyasu Sakane, Sachi Okabayashi, Shunsuke Kimura, Daisuke Inoue, Akiko Tanaka, Tomoyuki Furubayashi

**Affiliations:** 1 Department of Pharmaceutical Technology, Kobe Pharmaceutical University, Motoyamakita-machi 4-19-1, Higashinada, Kobe, Hyogo 658-8558, Japan; a-tanaka@kobepharma-u.ac.jp (A.T.); t-furu@kobepharma-u.ac.jp (T.F.); 2 New Drug Research Center Inc., Minatojimaminami-machi 7-1-14, Chuo-ku, Kobe, Hyogo 650-0047, Japan; s-okabayashi@ndrcenter.co.jp; 3 Department of Pharmacokinetics, Faculty of Pharmaceutical Sciences, Doshisha Women’s College of Liberal Arts, Kodo, Kyotanabe, Kyoto 610-0395, Japan; shkimura@dwc.doshisha.ac.jp; 4 College of Pharmaceutical Sciences, Ritsumeikan University, Noji-Higashi 1-1-1, Kusatsu, Shiga 525-8577, Japan; d-inoue@fc.ritsumei.ac.jp

**Keywords:** nose to brain delivery, monkey, species difference, nasal cavity, olfactory bulb, cribriform plate

## Abstract

Based on structural data on the nasal cavity and brain of the cynomolgus monkey, species differences in the olfactory bulb and cribriform plate were discussed from the viewpoint of direct delivery from the nose to the brain. Structural 3D data on the cynomolgus monkey skull were obtained using X-ray computed tomography. The dimensions of the nasal cavity of the cynomolgus monkey were 5 mm width × 20 mm height × 60 mm depth. The nasal cavity was very narrow and the olfactory region was far from the nostrils, similar to rats and humans. The weight and size of the monkey brain were 70 g and 55 mm width × 40 mm height × 70 mm depth. The olfactory bulb of monkeys is plate-like, while that of humans and rats is bulbar, suggesting that the olfactory area connected with the brain of monkeys is narrow. Although the structure of the monkey nasal cavity is similar to that of humans, the size and shape of the olfactory bulb are different, which is likely to result in low estimation of direct delivery from the nose to the brain in monkeys.

## 1. Introduction

The efficient delivery of drugs to the brain is very important for drug therapy for brain disorders such as Parkinson’s and Alzheimer’s disease, multiple sclerosis, and autism spectrum disorder. In general, the blood–brain barrier (BBB) strictly inhibits the entry of hydrophilic drugs into the brain from systemic circulation after intravenous and oral administration. The BBB is a continuous cerebral vasculature with endothelial cells connected by tight junctions formed by cell adhesion proteins, including occludin. Endocytosis activity is very low in the cerebral vasculature [1]. These features result in the failure of brain disorder treatment by hydrophilic and high molecular weight drugs such as peptides.

Some reviews have summarized strategies to overcome poor systemic drug delivery to the brain [2]. One of them is a direct delivery route from the nasal cavity. Small drugs, peptides, and even genes and cells were reported to be delivered directly to the brain after nasal application in rats. In our previous manuscripts, it was clarified that direct delivery from the nose to the brain (DDNB) enables two peptide drugs, oxytocin (MW: 1008 Da) [3] and CPN-116 (MW: 835 Da) [4] to act on the brain. Han et al. [5] showed that the concentration of plasmid DNA (7.2 kbase) in the brain after nasal administration was 10 times higher than the serum concentration, while after intravenous administration it was two orders of magnitude lower in the brain than that in the serum. After nasal application of plasmid DNA encoding β-galactosidase, a significantly higher expression was observed. According to Danielyan et al. [6], after mesenchymal stem cells and T406 human glioma cells stained with fluorescent dye are administered nasally, they can be observed in the thalamus, hippocampus, and cerebral cortex. After nasal administration of 3 × 10^5^ mesenchymal stem cells, 1000 cells were observed in the whole brain, indicating that 3% were successfully delivered. Similar results were described by Yu-Taeger et al. [7] and Galeano et al. [8].

In primary research, mice and rats have typically been used for in vivo studies on DDNB. However, the anatomy of the nasal cavity and brain and their connections in humans are different from those in animals. The data derived from animal studies should be carefully interpreted for extrapolation to humans. At the preclinical stage of the drug development process, monkeys are usually used to evaluate the pharmacokinetics and efficacy of drug candidates. The nasal cavities and brains of monkeys are expected to be more similar to those of humans than those of rats and mice. Invasive treatment is impossible in human studies, but determination of drug concentrations in the monkey brain is feasible. However, no manuscript has reported on the anatomy of the monkey brain and nasal cavity, while information on those of humans and rats is available [9,10,11,12]. In this study, to better understand the anatomy of the monkey nasal cavity, brain, and olfactory bulb, 3D data on the head of a cynomolgus monkey were obtained using X-ray computed tomography. Together with observations of the brain and the olfactory bulb, the anatomy of the brain and the nasal cavity of humans, monkeys, and rats was compared, and the influence of species differences on DDNB is discussed.

## 2. Materials and Methods

The skull and brain of the female monkey (5 years old) were provided by the New Drug Research Center, Inc. (Kobe, Japan). The head was taken from the monkey euthanized after another animal study by intramuscular ketamine and intravenous sodium pentobarbital followed by exsanguination. The animal study (study No: 18905) was approved on 24 October 2018 by the Animal Care Council at the New Drug Research Center, Inc. (approval No.: 181019A). The study was conducted in accordance with relevant national legislation.

The acquisition of 3D data of the monkey skull was outsourced to JMC Corporation (Yokohama, Kanagawa, Japan), in which an industrial nanofocus CT scanner, Phoenix Nanotom M (GE Measurement & Control, Tokyo, Japan) was used. Images were obtained under the setup of the CT scanner, as indicated below.
Voxel size:0.04 mm × 0.04 mmImage number:2000Accelerating voltage:140 kVFilament current:120 mA

The 3D data were visualized with VGSTUDIO MAX 3.2 (Volume Graphics GmbH, Heidelberg, Germany).

## 3. Results

### 3.1. Structure and Size of the Cynomolgus Monkey Nasal Cavity

**Figure 1:**
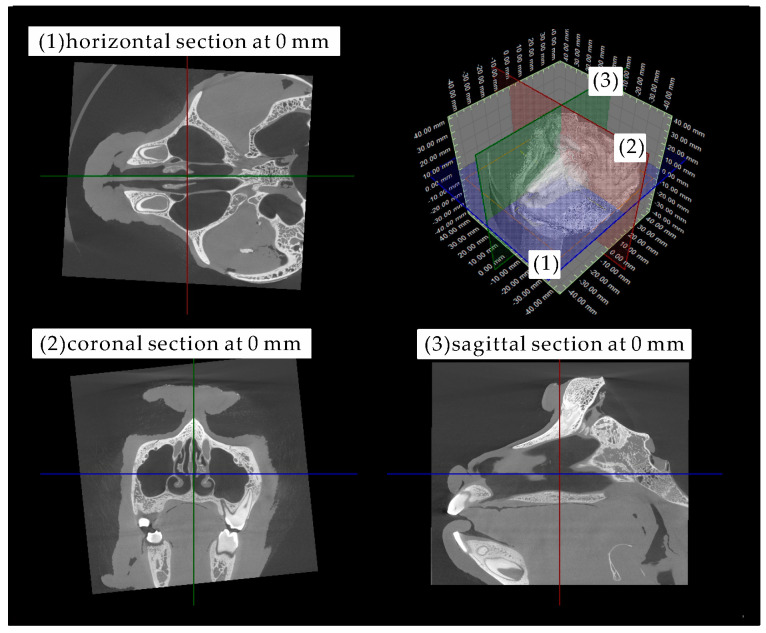
Morphology of the head of the cynomolgus monkey and the position criteria (0 mm) for the (**1**) horizontal (blue), (**2**) coronal (red), and (**3**) sagittal (green) sections.

Figure 1 shows the head of the cynomolgus monkey and the position criteria (0 mm) for the horizontal, coronal, and sagittal sections.

**Figure 2:**
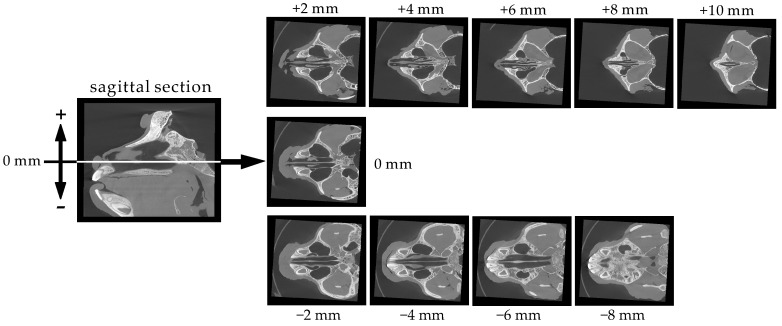
Serial horizontal sections (2 mm intervals) of the cynomolgus monkey skull.

Figure 2 shows horizontal sections at 2 mm intervals from the bottom (−8 mm) to the top (+10 mm) of the nasal cavity, which is very narrow. The paranasal sinus was confirmed to be a large round-shaped space just beside the nasal cavity. The sections from −8 to +10 mm indicate that the height of the nasal cavity is approximately 20 mm. According to the section at −6 mm, the bottom of the nasal cavity is wide (4 mm width). With increasing horizontal position, the nasal cavity becomes narrower. The sections at +2 mm, +4 mm, and +6 mm demonstrate that the structure of the upper part of the nasal cavity is complicated. This complication is likely due to nasal turbinates.

**Figure 3:**
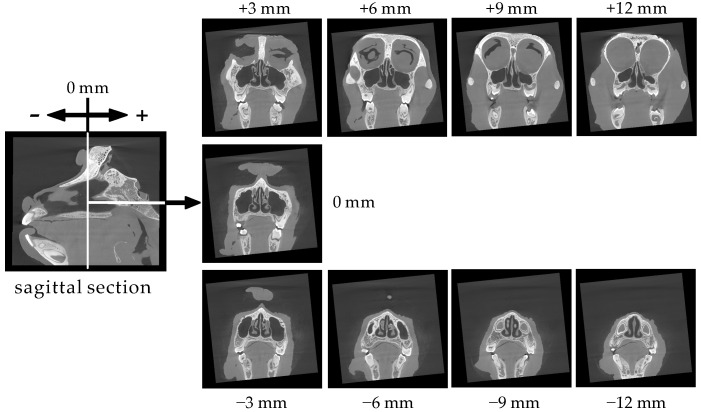
Serial coronal sections (3 mm intervals) of the cynomolgus monkey skull.

Figure 3 shows coronal sections at 3 mm intervals from the nostril to the pharynx. Complex structures due to the nasal turbinates are observed in the six sections from −9 to +6 mm.

**Figure 4:**
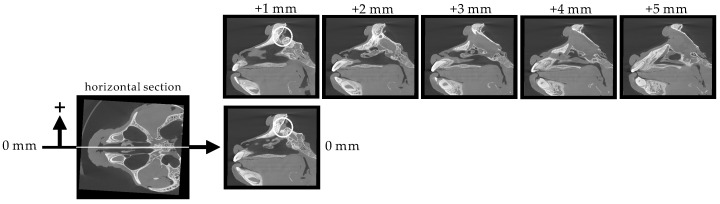
Serial sagittal sections (1 mm intervals) of the cynomolgus monkey skull.

Figure 4 shows sagittal sections of the nasal cavity at 1 mm intervals from the nasal septum (0 mm) to the side wall (+5 mm). These sections show that the width of the nasal cavity is 5 mm. It is noteworthy that a space extends from the brain to the upper right part of the nasal cavity (indicated by white circles). The space is visible in the 0 mm and +1 mm sections, but disappears in the +2 mm section, suggesting that it is very narrow (less than 2 mm wide).

### 3.2. Size of the Nasal Cavity and Relative Position with the Brain

**Figure 5:**
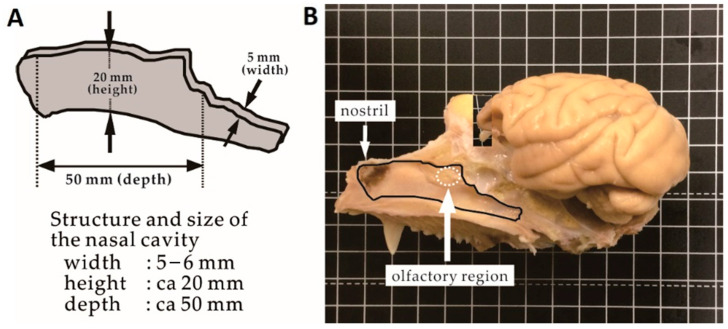
Scheme representing the structure and dimensions of the nasal cavity (**A**) and the locations of the nasal cavity and brain in the skull (**B**).

The left part of Figure 5 shows the shape and size of the nasal cavity. As demonstrated by the structural data above, the dimensions of the nasal cavity are 5–6 mm width, 20 mm height, and 50 mm depth. These data also indicate that the olfactory region, the area around the cribriform plate connected to the olfactory bulb, is 40 mm away from the nostril. Efficient delivery of drugs to the olfactory area using a normal spraying device is likely difficult. On the right side of Figure 5, the sagittal cross-section of the monkey skull is shown. The dissected brain was placed at the original position to show the locations of the nasal cavity and brain. As shown in the photo, the brain is located at the upper right position of the nasal cavity.

### 3.3. Size of the Brain

**Figure 6:**
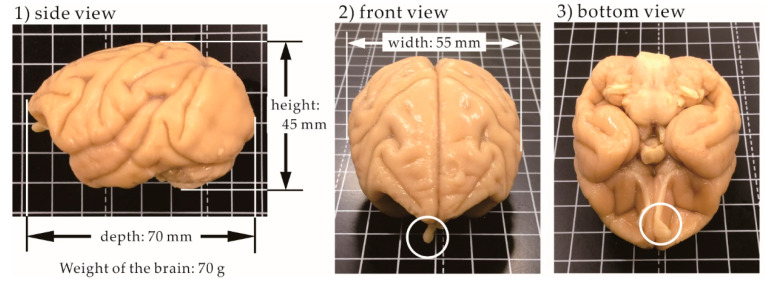
Photos of the cynomolgus monkey brain taken from three different angles.

Photos of the monkey brain taken from different angles are shown in Figure 6. The weight of the brain is 70 g. The dimensions of the brain are 45 mm height, 55 mm width, and 70 mm depth. The white circles indicate the right olfactory bulb. In the front view, the olfactory bulb has a plate-like structure. In the bottom view, the long olfactory tract connecting the olfactory bulb to the brain is shown. The appearance of the olfactory bulb is different from that of rats and humans.

## 4. Discussion

Monkeys have been used for some relevant in vivo studies. According to Kumar et al., after nasal spraying of progesterone, the concentration in the cerebrospinal fluid (CSF) in female rhesus monkeys was significantly higher than that after intravenous injection, although there were similar plasma concentrations [13]. A prostaglandin D agonist exhibited more potent pharmacological activity (sleep-inducing effect) in monkeys after nasal administration than after intravenous administration [14]. In the cynomolgus monkey, nasally administered interferon-β1b reached levels one order of magnitude higher in the olfactory bulb and trigeminal nerve than in other peripheral tissues. The brain distribution of interferon-β1b was visualized by autoradiography [15]. Iwasaki et al. investigated species differences in DDNB [16]. They used the ratio *K*p,in/*K*p,iv as an index of DDNB and clarified that the delivery of six compounds to the olfactory tract and the rest of the brain after nasal administration to monkeys was higher than that in rats, while the uptake by the olfactory bulb and trigeminal nerve was lower. The authors suggested that no mechanism offers better delivery in monkeys. A rare case of a human pharmacokinetic study was reported by Born et al. [17]. MSH/ACTH(4–10), vasopressin, and insulin were nasally applied to human volunteers, and the peptide concentrations in the lumbar CSF and serum were measured. There were much higher concentrations of MSH/ACTH(4–10) in the CSF after a 5 mg dose. Serum levels after 1 mg and 5 mg doses were similar to those in the placebo group.

Two cranial nerves connected to the nasal cavity, the olfactory and trigeminal nerves, are involved in the direct pathway from the nose to the brain [18]. The olfactory nerve extends from the olfactory bulb through small holes in the cribriform plate to the olfactory epithelium in the nasal cavity. Olfactory receptors are expressed at the terminal membranes of olfactory neurons. The role of the olfactory nerve is to transmit information on smells captured by olfactory receptors. The pathway in which the olfactory nerve is involved is called the olfactory route, through which it is feasible to deliver drugs to the cerebrum. In contrast, the trigeminal nerve extends from the pons to the nasal cavity. The route of the trigeminal nerve from the pons through the skull to the nasal cavity is much longer than that of the olfactory nerve. Drugs can be delivered directly to the pons and cerebellum through the trigeminal route. For this pathway, it is not yet clear how or through which route drugs are transported to the pons and cerebellum. Because the targets of many drugs acting on the brain are located in the cerebrum, this research focuses on the olfactory pathway.

**Figure 7:**
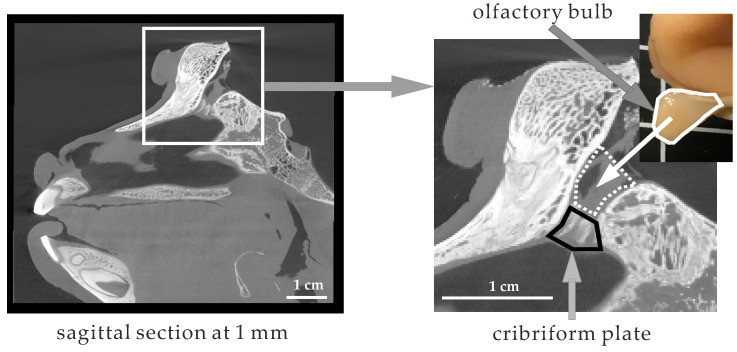
Location of the olfactory bulb and cribriform plate in the cynomolgus monkey skull.

Figure 7 shows the location of the olfactory bulb and cribriform plate in the monkey skull. The sagittal section at 1 mm is shown on the left side. The connection between the nasal cavity and brain is indicated by a white square. On the right side, the section in the white square is expanded. The narrow space, indicated by a white dashed line, is shown in the skull. Based on its shape, the narrow space fits the olfactory bulb, as the inset photo shows. The cribriform plate is located between the olfactory bulb and the nasal cavity. The thickness of the cribriform plate is likely 3–5 mm. The size of the cribriform plate is a determinant of the efficiency of DDNB. Judging from the shape of the olfactory bulb, the area of the cribriform plate of the monkey is likely small, leading to the underestimation of DDNB.

**Figure 8:**
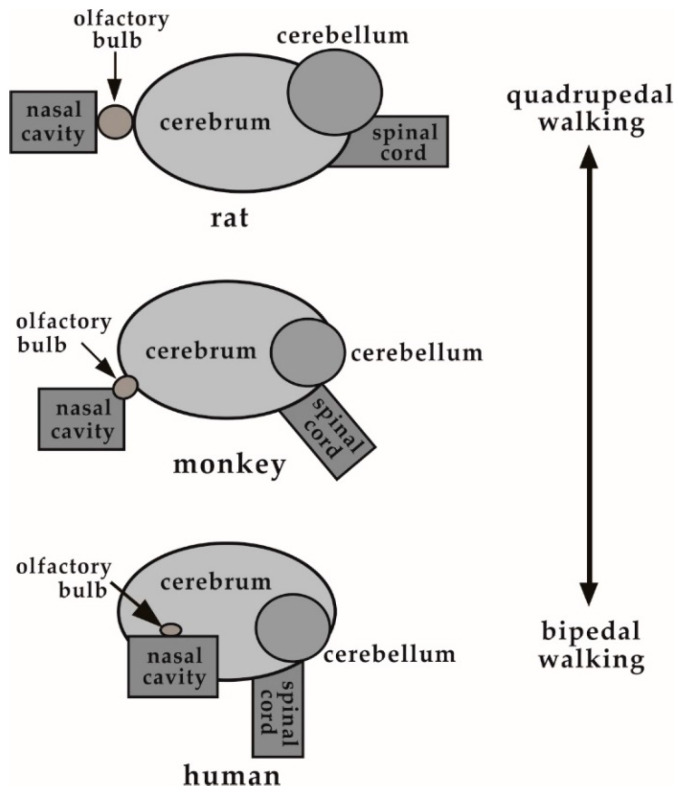
Changes in the relative locations of the nasal cavity, olfactory bulb, and brain in rats, monkeys, and humans.

Figure 8 shows a schematic representation of the locations of the nasal cavity and brain in rats, monkeys, and humans. In rats, walking quadrupedally, the nasal cavity is located in front of the brain and the spinal cord extends horizontally. In contrast, in humans, walking bipedally, the nasal cavity is translocated below the brain and the spinal cord extends vertically downward. The shift in the position of the nasal cavity relative to the brain likely represents evolutionary changes between rats and humans. The relative position of the nasal cavity in the monkey, walking bipedally at some times and quadrupedally at others, is possibly an intermediate between rats and humans.

## 5. Conclusions

The anatomy of the monkey nasal cavity is similar to that of humans and rats. The nasal cavity is narrow, and the olfactory region is away from the nostril. However, the olfactory bulb is different. The shape of the monkey olfactory bulb is plate-like, while that of humans and rats is bulbar. The difference suggests the small area of the olfactory region, possibly resulting in the underestimation of DDNB as compared to humans.
